# PIM3-AMPK-HDAC4/5 axis restricts MuERVL-marked 2-cell-like state in embryonic stem cells

**DOI:** 10.1016/j.stemcr.2022.08.009

**Published:** 2022-09-22

**Authors:** Xin Zhao, Jian Shen, Xuan Zhao, Miao Zhang, Xiao Feng, Weiyu Zhang, Xinyi Lu

**Affiliations:** 1 State Key Laboratory of Medicinal Chemical Biology, Nankai University, Tianjin 300350, People’s Republic of China

**Keywords:** embryonic stem cells, 2-cell-like cell, MuERVL, HDAC4/5, endogenous retrovirus, Pim3, AMPK, totipotency

## Abstract

A minority of embryonic stem cells (ESCs) marked by endogenous retrovirus MuERVL are totipotent 2-cell-like cells. However, the majority of ESCs repress MuERVL. Currently, it is still unclear regarding the signaling pathway(s) repressing the MuERVL-associated 2-cell-like state of ESCs. Here, we identify the PIM3-downstream signaling axis as a key route to repress MuERVL and 2-cell-like state. Downregulation, deletion, or inhibition of PIM3 activated MuERVL, 2-cell genes, and trophectodermal genes in ESCs. By screening PIM3-regulated pathways, we discovered AMPK as its key target. The loss of *Pim3* caused an increase in AMPK phosphorylation, which phosphorylated HDAC4/5 and triggered their transfer out of the nucleus in *Pim3*^−/−^ ESCs. The reduction of nuclear HDAC4/5 caused increased H3K9ac and reduced H3K9me1/2 enrichment on MuERVL, thus activating MuERVL and 2-cell-like state. In summary, our study uncovers a novel axis by which PIM3 suppresses 2-cell marker MuERVL and totipotent state in ESCs.

## Highlights

• PIM3 signaling pathway represses MuERVL and 2-cell-like state
• *Pim3* loss promotes AMPK phosphorylation, which activates MuERVL
• Phosphorylated AMPK mediates HDAC4/5 export from the nucleus
• HDAC4/5 repress MuERVL through modulating H3K9ac and H3K9me1/2

## Graphical abstract

**Figure:**
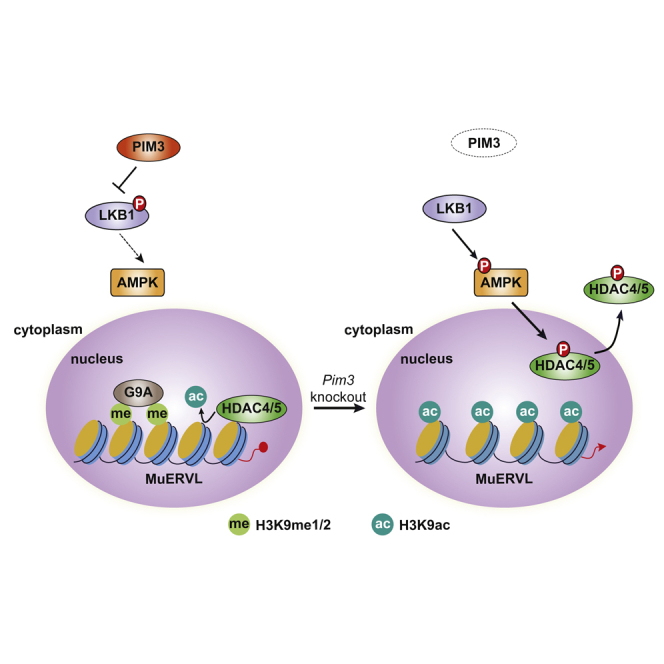

## Teaser

Zhao et al. discover the PIM3 signaling pathway as a key route to repress MuERVL and 2-cell-like totipotent state in ESCs. *Pim3* loss allows phosphorylation of AMPK, which phosphorylates HDAC4/5 and promotes their transport out of the nucleus, thereby permitting acetylation of H3K9 and interfering with H3K9me1/2 deposition on MuERVL. Their findings provide a novel strategy to activate 2-cell-like totipotency.

## Introduction

Mouse embryonic stem cells (ESCs) are pluripotent and can differentiate into three germ layers, but they cannot contribute to extraembryonic tissues when they are injected into blastocyst (Beddington and Robertson, 1989). A small percentage of cells within the ESC population, named 2-cell (2C)-like cells, can contribute to both inner cell mass and extraembryonic cells, including the trophectoderm of the blastocyst (Macfarlan et al., 2012). These cells highly express endogenous retrovirus MuERVL, which marks 2C embryos and has been used as a marker to determine the 2C-like totipotent state of ESCs (Macfarlan et al., 2012). In ESCs, MuERVL is usually silenced transcriptionally and epigenetically. The absence of epigenetic factors, such as LSD1, ZMYM2, and FACT, activates 2C-like state in ESCs (Chen et al., 2020; Macfarlan et al., 2011; Yang et al., 2020). Alternatively, the 2C-like state of ESCs can also be activated by ectopically expressing transcription activators of MuERVL, for example, DUX, ZSCAN4, and DPPA2/4 (De Iaco et al., 2017, 2019; Hendrickson et al., 2017; Whiddon et al., 2017; Yan et al., 2019; Zhang et al., 2019). Recently, it was found that the retinoic acid signaling pathway and the P53 pathway are able to induce 2C-like fate (Grow et al., 2021; Iturbide et al., 2021; Tagliaferri et al., 2016, 2019). However, it is still unknown whether there is any signaling pathway to repress 2C-like totipotent state and MuERVL in ESCs.

An important signaling pathway in ESCs is the PIM signaling pathway. There are three highly conserved members (PIM1, PIM2, and PIM3) within the PIM family of serine/threonine kinases (Santio and Koskinen, 2017). The catalytic domains of PIM kinases are highly identical (∼60%) to each other as well (Nawijn et al., 2011). They participate in cell cycle regulation, energy metabolism, cell proliferation, and apoptosis (Beharry et al., 2011; Morishita et al., 2008; White, 2003). PIM family members are constitutively active; therefore, their kinase activities are positively correlated with their transcriptional levels (Lilly et al., 1999; Qian et al., 2005). However, they demonstrate tissue-specific expression patterns (Eichmann et al., 2000; Meeker et al., 1987). Different PIM kinases also exhibit distinct functions despite their protein sequence similarities (Nawijn et al., 2011), suggesting that each PIM kinase may behave differently. The expression of PIM kinases can be activated by the LIF signaling pathway and downstream JAK-STAT (Aksoy et al., 2007; Mary Photini et al., 2017), which are important to ESC pluripotency maintenance (Cartwright et al., 2005; Raz et al., 1999). These findings suggest a potential functional role of PIM kinases in ESCs.

In this study, we set out to screen the PIM kinases with shRNAs and small molecules to identify their potential role in regulating potency expansion in ESCs. We highlight PIM3-AMPK-HDAC4/5 axis as a novel path to prevent ESCs from entering the 2C-like state.

## Results

### Screening of PIM kinases for the repressor of 2C marker MuERVL

**Figure 1:**
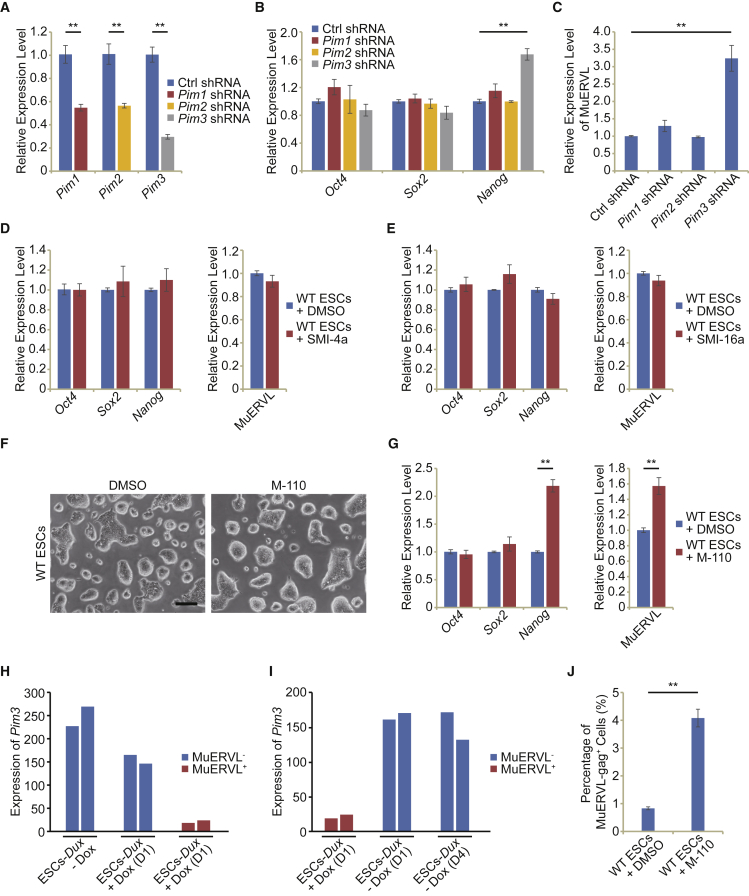
Depletion of PIM kinases by shRNAs and inhibitors (A) qPCR analysis of the expression of *Pim1*, *Pim2*, and *Pim3* after the depletion of *Pim* family members in ESCs respectively. (B) qPCR analysis of the expression of pluripotency markers (*Oct4*, *Sox2*, and *Nanog*) after the depletion of *Pim* family members in ESCs. (C) The expression level of MuERVL determined by qPCR in ESCs treated with control (Ctrl) shRNA or *Pim* shRNAs. (D) qPCR analysis of the expression of pluripotency markers and MuERVL in control ESCs and ESCs treated with the inhibitor of PIM1 kinase (SMI-4a). DMSO-treated sample was included as a control. (E) The expression levels of pluripotency markers and MuERVL in ESCs treated with DMSO and the inhibitor of PIM1/2 kinases (SMI-16a), respectively, as measured by qPCR and normalized to *Gapdh* level. (F) Cell morphology of WT ESCs treated with an inhibitor of PIM3 kinase (M−110). DMSO-treated samples were included as control. Scale bar, 100 μm. (G) qPCR analysis of the expression of pluripotency markers and MuERVL in control ESCs and ESCs treated with M-110. All qPCR data in Figure 1 are biological triplicate data (n = 3 independent experiments) and are presented as mean ± SEM. (H and I) The RNA-seq expression level (cpm) of *Pim3* during the entry to (H) and exit from (I) 2C-like state in the ESCs expressing dox-induced *Dux*, which drives ESCs to 2C-like state. + dox, addition of dox; – dox, removal of dox; D1, day 1; D4, day 4. (J) Flow cytometry analysis of the MuERVL-gag^+^ population in control ESCs and ESCs treated with M-110. DMSO-treated samples were added as control. Biological triplicate data (n = 3 independent experiments) are presented as mean ± SEM; ^∗∗^p < 0.01 in Student’s t test.

Since the activities of PIM kinases are dependent on their expression (Lilly et al., 1999; Qian et al., 2005), we examined their mRNA levels by qPCR in ESCs and after differentiation (Figures S1A–S1C). Among three PIM kinases, *Pim3* was expressed highest in ESCs, and its expression was close to that of pluripotency genes (*Oct4*, *Sox*2, and *Nanog*) (Figure S1A). During ESC differentiation induced by LIF withdrawal, *Pim1* and *Pim2* expression remained unchanged or upregulated, but the expression of *Pim3* was downregulated, similar to that of pluripotency genes (Figures S1B and S1C), implying that *Pim3* is required to specifically express at a high level in ESCs. Interestingly, *Pim3* downregulation was accompanied by the activation of 2C-marker MuERVL after LIF withdrawal (Figure S1D). Upon directed differentiation of ESCs to trophectodermal stem cells (TSCs), only *Pim3* was downregulated, whereas the expression of *Pim1* and *Pim2* was upregulated (Figures S1E and S1F), implying that *Pim3* is not required to be expressed in TSCs. To study the role of PIM kinases in regulating 2C-like totipotent state, we examined the expression of 2C-marker MuERVL after depleting the expression of *Pim1/2/3* with shRNAs (Figure 1A). The depletion of *Pim1*, *Pim2*, or *Pim3* did not affect *Oct4* and *Sox2* expression except that *Nanog* was activated by *Pim3* depletion (Figure 1B). Intriguingly, the depletion of *Pim3*, but not *Pim1* or *Pim2*, activated the expression of MuERVL and MuERVL-marked 2C-like state (Figures 1C and S1G). Furthermore, the addition of a small molecule inhibitor of PIM1 (SMI-4a) (Lin et al., 2010) or the inhibitor of PIM1 and PIM2 (SMI-16a) (Xia et al., 2009) to ESC culture did not influence the expression of pluripotency genes and MuERVL (Figures 1D and 1E), confirming the results that PIM1 or PIM2 activity is not required for ESC pluripotency or MuERVL suppression. However, similar to *Pim3* depletion, inhibition of PIM3 kinase activity with M−110 evoked the activation of MuERVL and *Nanog* without altering ESC morphology (Figures 1F and 1G). The effects of M−110 on MuERVL and 2C genes were confirmed in another ESC line J1 (Figures S1H and S1I). We further confirmed the role of PIM3 in the alternation of ESC fate by examining *Pim3* expression during the natural transition between ESCs and 2C-like cells according to the analysis of published RNA-seq data (Fu et al., 2019, 2020). We found that *Pim3* was repressed in MuERVL^+^ 2C-like cells, while it was re-activated upon exit from 2C-like state (Figures 1H and 1I), suggesting the implication of PIM3 in the conversion to 2C-like state. In line with these findings, the percentage of MuERVL-gag^+^ 2C-like cells increased after treating ESCs with PIM3 inhibitor M−110 (Figure 1J). These results suggest that PIM3, which lies at the downstream of LIF signaling, acts as a key repressor of MuERVL and 2C genes in ESCs.

### *Pim3*-deficient ESCs activate MuERVL and 2C-like totipotent state

**Figure 2:**
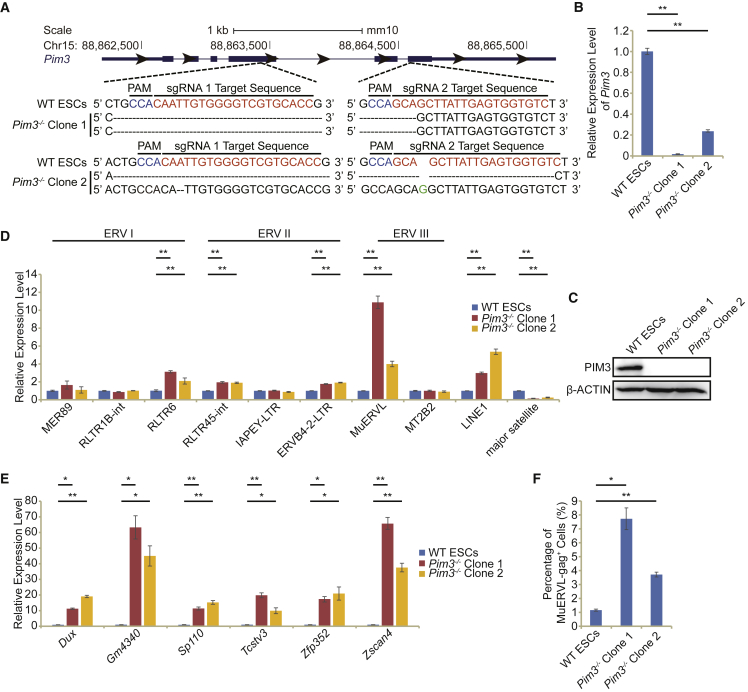
Establishment of *Pim3*-deficient ESCs (A) Schematic of mutation sites in two *Pim3*^−/−^ ESC clones. Black dashes: deleted bases; green base: insertion; red bases: sgRNA target sequences; blue bases: protospacer adjacent motif (PAM) sequences. (B) qPCR analysis of the expression of *Pim3* in WT ESCs and *Pim3*^−/−^ ESCs. (C) Western blot analysis of PIM3 protein in WT ESCs and *Pim3*^−/−^ ESCs. β-ACTIN was used as a loading control. (D) qPCR analysis of the expression of retrotransposons and other repeats in WT ESCs and *Pim*3^−/−^ ESCs. (E) qPCR analysis of the expression of 2C embryo genes in WT ESCs and *Pim*3^−/−^ ESCs. All qPCR data in Figure 2 are biological triplicate data (n = 3 independent experiments) and are presented as mean ± SEM. (F) Flow cytometry analysis of the MuERVL-gag^+^ population in WT ESCs or *Pim*3^−/−^ ESCs. Biological triplicate data (n = 3 independent experiments) are presented as mean ± SEM; ^∗^p < 0.05, ^∗∗^p < 0.01 in Student’s t test.

To further verify the role of *Pim3* in ESCs, we generated *Pim3* knockout cell lines. We designed two guide RNAs targeting the exon 4 and exon 6 of *Pim3* gene, respectively, and generated two *Pim3* knockout (*Pim3*^−/−^) ESC lines (Figure 2A and S2A). qPCR analysis showed the successful deletion of *Pim3* fragments (Figures 2B and S2B). The western blot demonstrated the complete loss of PIM3 protein in *Pim3*^−/−^ ESCs (Figure 2C). *Pim3* deficiency did not disrupt ESC morphology and expression of pluripotency genes (*Oct4* and *Sox2*), although its absence stimulated the upregulation of *Nanog* (Figures S2C–S2E). NANOG activation appeared to prevent further activation of MuERVL in *Pim3*^−/−^ ESCs (Figures S2F and S2G). The downregulation of *Nanog* resulted in the activation of MuERVL in wild-type (WT) ESCs (Figure S2F). Depletion of *Nanog* further activated MuERVL expression in *Pim3*^−/−^ ESCs (Figure S2G). Consistent with results from *Pim3* depletion and inhibition, loss of *Pim3* resulted in the upregulation of MuERVL and some other ERVs, including RLTR6, RLTR45-int, and LINE1 (Figure 2D). 2C genes, such as *Dux*, *Gm4340*, *Sp110*, *Tcstv3*, *Zfp352*, and *Zscan4*, were similarly activated in *Pim3*^−/−^ ESCs (Figure 2E). In addition, *Pim3* deletion caused MuERVL-gag^+^ 2C-like totipotent cell population in ESCs to elevate from ∼1% to 4%–8% (Figures 2F and S2H). These results confirm the PIM3-mediated repression of MuERVL and 2C-like state, which were suppressed by NANOG as well.

### Transcriptome analysis reveals PIM3-target genes

**Figure 3:**
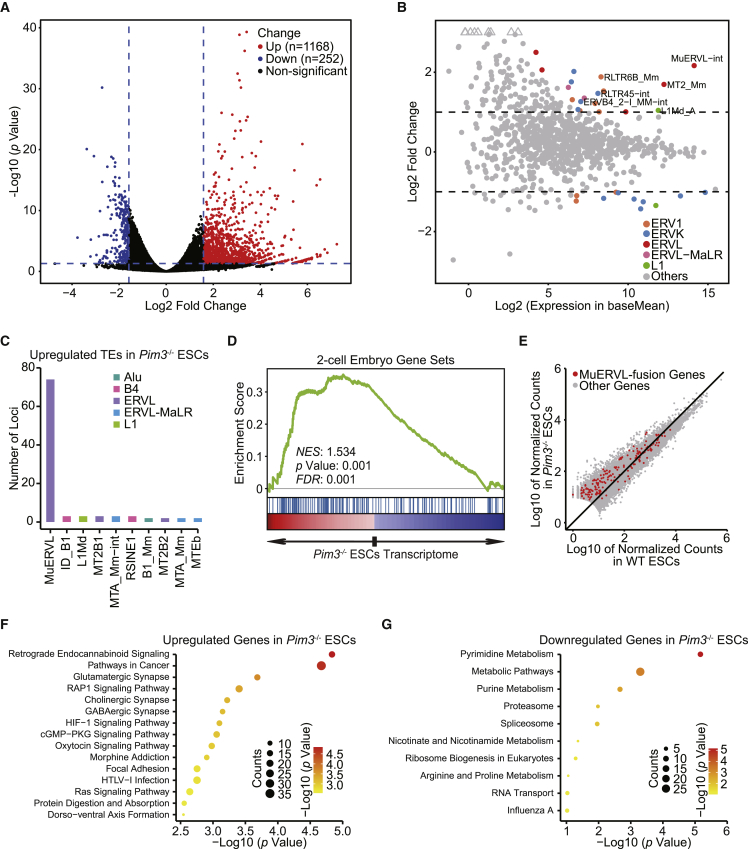
Transcriptome profile regulated by *Pim3* (A) The volcano plot of gene expression in *Pim*3^−/−^ ESCs versus WT ESCs. Red, upregulated genes; blue, downregulated genes. Genes with expression change ≥ 3-fold and adjusted p < 0.05 are shown. (B) A scatter diagram shows a transcriptome analysis of TE expression after *Pim3* knockout. The result from Squire was used to plot the diagram. Different colored dots represent different retroelements. Colored dots indicate TEs with significant expression change (p < 0.05, Wald test). Triangles represent TEs with log_2_ (fold change) > 4. (C) The top 10 TEs with the highest number of loci upregulated after *Pim3* knockout. (D) Gene set enrichment analysis (GSEA) of 2C genes in the transcriptome of *Pim3*^−/−^ ESCs. Red, upregulated genes; blue, downregulated genes; *NES*, normalized enrichment scores; *FDR*, false discovery rate. The Kolmogorov-Smirnov statistic was used for the calculation of p value. (E) Dot plot of all expressed genes in WT ESCs and *Pim*3^−/−^ ESCs. Genes with alternative transcripts overlapped with MuERVL are labeled in red. (F and G) KEGG terms of pathways related to upregulated genes (F) and downregulated genes (G) after *Pim3* knockout. The analysis was done with DAVID. Color gradient indicates significance in –log_10_ (p value), and the size of dots indicates the number of genes in the corresponding pathway.

Next, we further probed the function of *Pim3* by profiling transcriptome in two independent clone lines of *Pim3*^−/−^ ESCs. More genes were upregulated than downregulated in *Pim3*^−/−^ ESCs (Figure 3A; Data S1). The absence of *Pim3* caused activation of 2C-marker MuERVL and other transposable elements (TEs), as well as downregulation of ERVK family members (Figures 3B and S3A and Data S2). Moreover, the number of activated MuERVL loci was the highest among all activated TEs (Figures 3C and S3B). Since MuERVL marks the 2C state, *Pim3* deletion resulted in the upregulation of 2C genes and activation of MuERVL-chimeric genes in ESCs (Figures 3D and 3E), implying the activation of 2C-like state after *Pim3* loss. Principal component analysis (PCA) based on gene expression suggested a moderate shift of *Pim3*^−/−^ transcriptome toward that of 2C-like cells (Figure S3C), while 87 genes of 2C-like cells (Macfarlan et al., 2012) were activated in *Pim3*^−/−^ cells (Figure S3D). The majority of TEs that are activated in 2C embryos (Macfarlan et al., 2012) demonstrated activation in *Pim3*^−/−^ ESCs (Figure S3E), further supporting the transition of *Pim3*^−/−^ cells toward 2C-like cells. The partial transition toward 2C-like cells is probably because only a subset of *Pim3*^−/−^ ESCs transit to 2C-like cells, whereas the other cells remain in non-2C-like state. Consistent with the downregulation of *Pim3* in TSCs (Figures S1E and S1F), ESCs demonstrated upregulation of TSC genes (Lee et al., 2019) after *Pim3* deletion (Figure S3F). *Pim3* knockout led to the activation of signaling pathways that were related to cancer, HTLV-1 infection, and neural synapses, while its absence repressed pathways related to metabolism, proteasome, and spliceosome (Figures 3F and 3G). The downregulation of spliceosome activity was recently linked to the activation of totipotent state (Shen et al., 2021). These data support the idea that *Pim3* deficiency activates the MuERVL-marked 2C-like totipotent state and trophectodermal genes in ESCs.

### AMPK at the downstream of PIM3 activates MuERVL and 2C-like state

**Figure 4:**
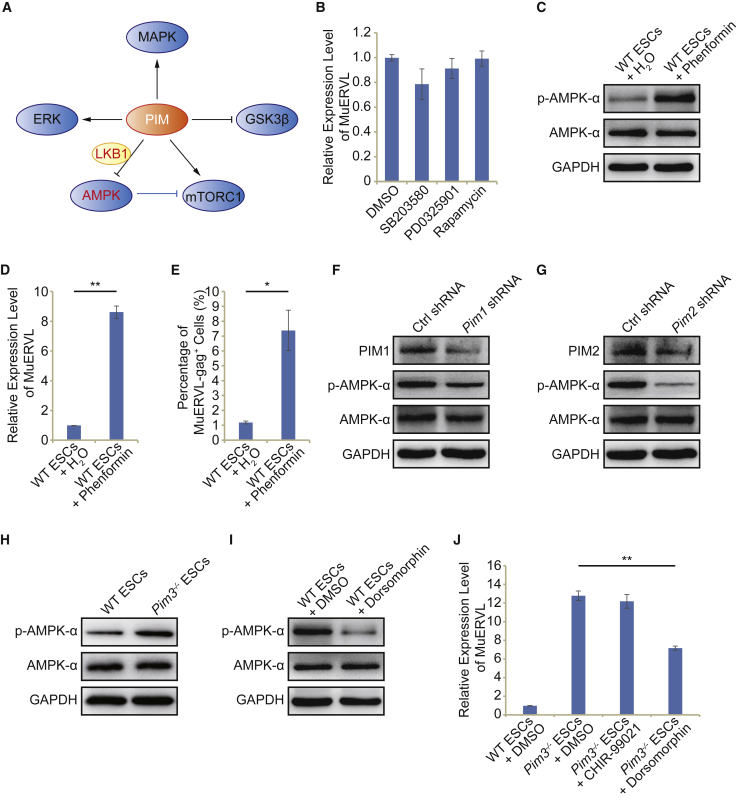
Screening of PIM3-related pathways identifies AMPK as a regulator of 2C-like totipotent state (A) Schematic diagram of signaling pathways involving PIM kinases. The blue objects represent downstream targets of PIM kinases. (B) qPCR analysis of the expression of MuERVL in control ESCs and ESCs treated with inhibitors. DMSO-treated samples were included as a control. (C) Western blot analysis of AMPK-α and *p*-AMPK-α proteins in WT ESCs treated with an activator of AMPK (phenformin). H_2_O treated samples were included as a control. GAPDH was used as a loading control. Alpha (α) refers to the alpha subunit of AMPK. (D) qPCR analysis of the expression of MuERVL in control ESCs and ESCs treated with 1.5 mM phenformin. (E) Flow cytometry analysis of the MuERVL-gag^+^ population in WT ESCs treated with water and phenformin respectively. Water-treated sample was included as a control. Biological triplicate data (n = 3 independent experiments) are presented as mean ± SEM. (F) Western blot analysis of the indicated proteins in WT ESCs expressing *Pim1* shRNA or control shRNA. GAPDH was included as a loading control. (G) Western blot analysis of the indicated proteins in WT ESCs expressing *Pim2* shRNA or control shRNA. GAPDH was included as a loading control. (H) Western blot analysis of AMPK-α and *p*-AMPK-α levels in WT ESCs and *Pim*3^−/−^ ESCs. GAPDH was used as a loading control. (I) Immunoblot analysis of AMPK-α and *p*-AMPK-α levels in WT ESCs treated with the inhibitor of AMPK (dorsomorphin). DMSO-treated sample was included as a control. GAPDH was used as a loading control. (J) qPCR analysis of the expression of MuERVL in WT ESCs and *Pim*3^−/−^ ESCs treated with dorsomorphin or CHIR-99021. DMSO-treated sample was included as a control. All qPCR data in Figure 4 are presented as mean ± SEM (n = 3 independent experiments). ^∗^p < 0.05, ^∗∗^p < 0.01 in Student’s t test.

To study how *Pim3* inadequacy activates 2C-like state in ESCs, we looked into the literature on the function of PIM kinases. It is found that PIM kinases activate MAPK (Leung et al., 2020; Santio et al., 2016), ERK (Narlik-Grassow et al., 2012), and mTORC1 (Beharry et al., 2011) pathways but suppress GSK3β (Narlik-Grassow et al., 2012) and AMPK pathways (Beharry et al., 2011; Mung et al., 2021) (Figure 4A). We validated the roles of these pathways in the PIM-mediated repression of MuERVL by treating ESCs with inhibitors of PIM-activated pathways or activators of PIM-repressed pathways. The inhibition of MAPK (with SB203580), ERK (with PD0325901), or mTORC1 (with rapamycin) did not perturb MuERVL expression (Figure 4B). In contrast, activation of the AMPK with phenformin efficiently released MuERVL from repression and induced the emergence of the MuERVL-gag^+^ 2C-like population in ESCs, phenocopying the consequences of *Pim3* deletion (Figures 4C–4E and S4A). It was previously reported that *Pim* deficiency in mouse fibroblast cells elicited increased AMPK phosphorylation (Beharry et al., 2011). Unexpectedly, the depletion of *Pim1* or *Pim2* led to the downregulation of AMPK phosphorylation (Figures 4F and 4G). This was confirmed with inhibitors of PIM1 and PIM2 (Figure S4B). In contrast, AMPK phosphorylation only rose in *Pim3*^−/−^ ESCs (Figure 4H). Furthermore, the inhibition of AMPK with dorsomorphin (Figure 4I), but not the inhibition of GSK3β with CHIR-99021, partially rescued MuERVL expression in *Pim3*^−/−^ ESCs (Figure 4J). Depletion of *Lkb1*, which lies at the downstream of PIM3 and mediates AMPK activation (Mung et al., 2021), partially rescued MuERVL expression as well (Figures S4C–S4E). Together, these results indicate that AMPK is a key downstream target of PIM3 in restraining MuERVL and 2C-like state.

### Phosphorylated AMPK relieves MuERVL and 2C-like state from the suppression of HDAC4/5

**Figure 5:**
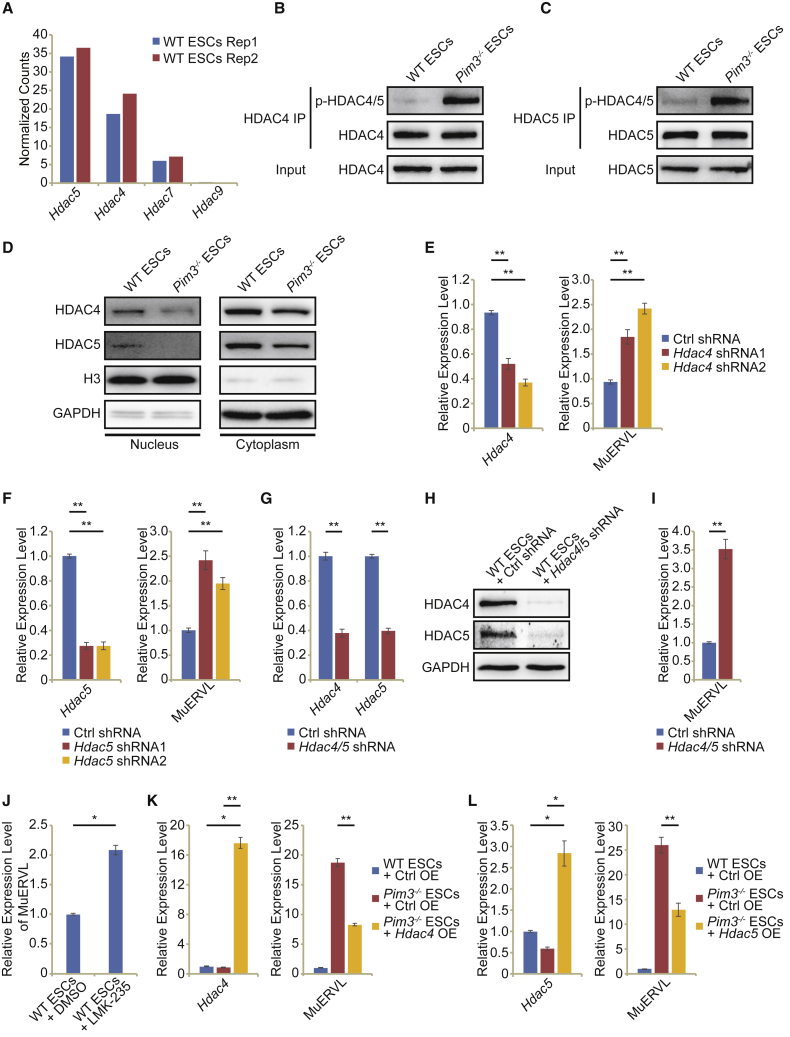
HDAC4/5 function at the downstream of AMPK to repress MuERVL-marked 2C-like totipotent state (A) The RNA-seq expression levels of class IIa HDACs (*Hdac4*, *Hdac5*, *Hdac7*, and *Hdac9*) in two replicates of WT ESCs. (B and C) Western blot analysis after immunoprecipitation with HDAC4 (B) or HDAC5 (C) antibody in WT ESCs and *Pim*3^−/−^ ESCs. Input was loaded as control. (D) Western blot analysis of HDAC4 and HDAC5 expression in the nucleus and cytoplasm of WT ESCs and *Pim*3^−/−^ ESCs. GAPDH was used as a loading control to the cytoplasm. H3 was used as a loading control to the nucleus. (E) qPCR expression levels of *Hdac4* and MuERVL after the depletion of *Hdac4* in ESCs. (F) qPCR expression levels of *Hdac5* and MuERVL after the depletion of *Hdac5* in ESCs. (G) qPCR expression levels of *Hdac4* and *Hdac5* after depleting *Hdac4/5* by shRNAs in ESCs. (H) Western blot analysis of the HDAC4 and HDAC5 in WT ESCs expressing *Hdac4/5* shRNAs or control (Ctrl) shRNA. GAPDH was added as a loading control. (I) qPCR analysis of the expression of MuERVL after the shRNA depletion of *Hdac4/5* in ESCs. (J) qPCR analysis of the expression of MuERVL in control ESCs and ESCs treated with an inhibitor of HDAC4/5 (LMK-235). DMSO-treated sample was included as a control. (K) The expression levels of *Hdac4* and MuERVL after *Hdac4* overexpression (OE) in WT ESCs and *Pim*3^−/−^ ESCs, as measured by qRT-PCR and normalized to *ACTB* level. (L) The expression levels of *Hdac5* and MuERVL after *Hdac5* overexpression in WT ESCs and *Pim*3^−/−^ ESCs, as measured by qRT-PCR and normalized to *ACTB* level. All qPCR data in Figure 5 are presented as mean ± SEM (n = 3 independent experiments). ^∗^p < 0.05, ^∗∗^p < 0.01 in Student’s t test.

A previous study showed that a key AMPK downstream target was class IIa HDACs (Mihaylova et al., 2011). Increased AMPK phosphorylation induced elevated phosphorylation of HDAC4/5 in hepatocytes (Mihaylova et al., 2011). This prompted us to explore the impact of *Pim3* deletion on class IIa HDACs. We first looked into the expression of class IIa HDACs (*Hdac4*, *Hdac5*, *Hdac7*, and *Hdac9*) in ESCs. *Hdac5* was expressed highest in ESCs, followed by *Hdac4*, whereas *Hdac7* and *Hdac9* remained lowly expressed or unexpressed (Figure 5A). Hence, we focused on studying HDAC5 and HDAC4. We immunoprecipitated HDAC4/5 respectively and found their phosphorylation increased after *Pim3* deletion (Figures 5B and 5C). The inhibition of AMPK with dorsomorphin reduced the phosphorylation of HDAC4/5 (Figure S5A), confirming HDAC4/5 as the downstream targets of AMPK. Since class IIa HDACs can shuttle between the nucleus and the cytoplasm, we extracted the nuclear and cytoplasmic proteins to test whether HDAC4/5 phosphorylation affects their localization. Indeed, the amount of nuclear HDAC4/5 was severely reduced in *Pim3*^−/−^ ESCs (Figure 5D). These findings suggest that HDAC4/5 lie at downstream of AMPK and PIM3 in ESCs.

We subsequently questioned whether the activation of 2C-like state and MuERVL resulted from the increased HDAC4/5 phosphorylation and their decreased nuclear localization. Therefore, we knocked down *Hdac4/5* separately and at the same time. Depletion of either *Hdac4* or *Hdac5* activated MuERVL expression (Figures 5E and 5F). The simultaneous depletion of both *Hdac4* and *Hdac5* further activated MuERVL expression (Figures 5G–5I). The phenotype of MuERVL activation and increased 2C-like population was recapitulated after treating ESCs with the HDAC4/5 selective inhibitor LMK-235 (Marek et al., 2013) (Figures 5J and S5B). Conversely, overexpression of HA-tagged HDAC4/5 in *Pim3*^−/−^ ESCs increased nuclear HDAC4/5 levels and partially rescued the expression of MuERVL (Figures 5K, 5L, and S5C–S5F), confirming HDAC4/5 as the functional targets of PIM3. Altogether, these findings reveal that AMPK mediates the export of HDAC4/5 out of the nucleus to relieve MuERVL and 2C-like totipotent state from silencing.

### PIM3 deficiency elevated H3K9ac and reduced H3K9me1/2 density on MuERVL

**Figure 6:**
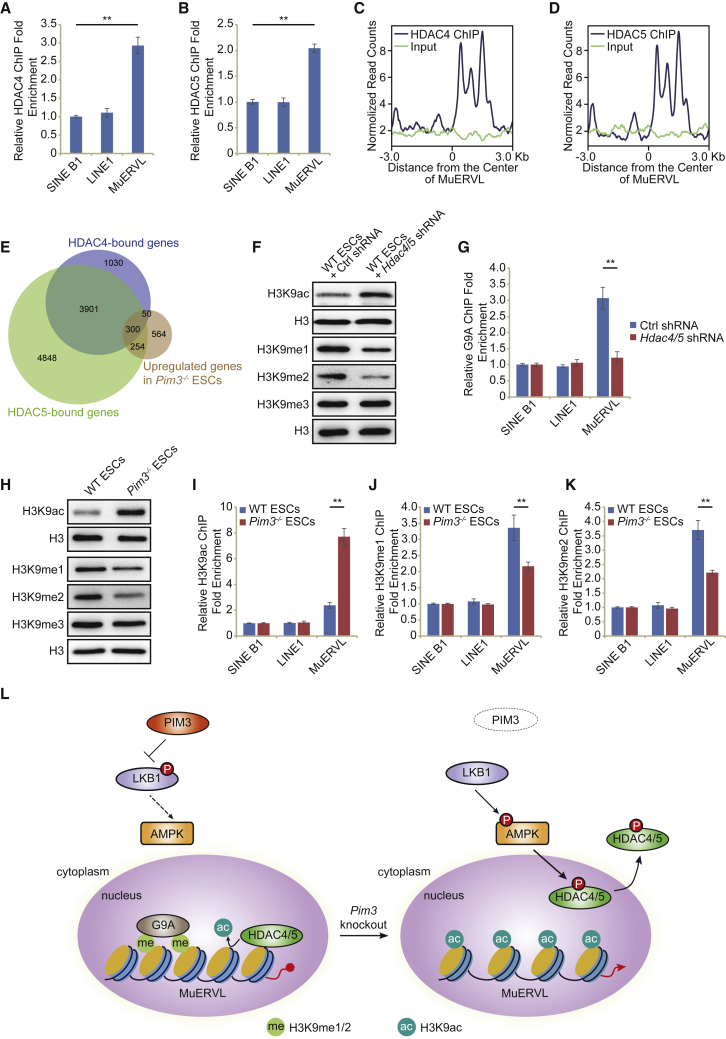
HADC4/5 repress MuERVL through H3K9me1/2 (A and B) ChIP-qPCR analysis of HDAC4 (A) and HDAC5 (B) binding on different retrotransposons. ChIP-qPCR data were normalized to input and that of the control region. (C and D) HDAC4 (C) and HDAC5 (D) binding profile around the center of MuERVL locus. The ChIP-seq signal was presented as normalized read counts. (E) The overlap between upregulated genes in *Pim*3^−/−^ ESCs and HDAC4/5 binding genes. (F) Western blot analysis of H3K9ac and H3K9me1/2/3 levels in WT ESCs expressing *Hdac4*/5 shRNA or control (Ctrl) shRNA. H3 was used as a loading control. (G) ChIP-qPCR analysis of G9A enrichment on MuERVL after shRNA depletion of *Hdac4/5* in ESCs. ChIP-qPCR data were normalized to input and SINE B1. (H) Western blot analysis of H3K9me1/2/3 and H3K9ac levels in WT and *Pim*3^−/−^ ESCs. H3 was used as a loading control. (I–K) ChIP-qPCR analysis of H3K9ac (I), H3K9me1 (J), and H3K9me2 (K) enrichment on MuERVL in WT ESCs and *Pim3*^−/−^ ESCs. ChIP-qPCR data were normalized to input and SINE B1. All ChIP-qPCR data in Figure 6 are biological triplicate data (n = 3 independent experiments) and are presented as mean ± SEM; ^∗∗^p < 0.01 in Student’s t test. (L) Schematic of PIM3 signaling pathway repressing 2C-like totipotent state in embryonic stem cells. PIM3 phosphorylates LKB1 and thereby inhibits its activity to phosphorylate AMPK, while hypo-phosphorylated HDAC4/5 remain in the nucleus to repress MuERVL and associated 2C-like state by deacethylating H3K9 and allowing G9A to deposit H3K9me1/2; in the absence of PIM3, *p*-AMPK phosphorylates HDAC4/5 and mediates their cytoplasmic localization, which promotes H3K9ac enrichment and H3K9me1/2 loss on MuERVL, and subsequent activation of MuERVL and 2C-like state from repression. The purple circle represents the nucleus. Dashed arrow: events that have not occurred.

To identify how HDAC4/5 modulate MuERVL and 2C-like state, we validated the binding of HDAC4/5 to MuERVL. ChIP-qPCR results suggested that HDAC4/5 directly interacted with and repressed MuERVL in ESCs (Figures 6A, 6B, 5K, and 5L). HDAC4/5 ChIP-seq data revealed that HDAC4/5 were mainly bound to the intergenic regions and introns in ESCs (Figures S6A and S6B). The bindings of HDAC4/5 were enriched on MuERVL (Figures 6C and 6D), suggesting the direct regulatory roles of HDAC4/5 on MuERVL expression. In addition, 51.71% (604/1168) of upregulated genes in *Pim3*^−/−^ ESCs were bound by HDAC4/5 (Figures 6E and 3A), suggesting a major role of HDAC4/5 in regulating downstream genes of PIM3. A possible way to activate MuERVL after *Pim3* loss is through activating 2C genes, which are known to promote MuERVL expression (De Iaco et al., 2017; Hendrickson et al., 2017; Whiddon et al., 2017). Indeed, we observed the binding of HDAC4/5 to the *Dux* promoter region and the activation of *Dux* in *Pim3*^−/−^ ESCs (Figures 2E and S6C). Depletion of *Dux* partially rescued the activation of MuERVL in *Pim3*^−/−^ ESCs (Figure S6D), suggesting that there are other ways to activate MuERVL besides DUX. MuERVL is known to be repressed by H3K9me2 as well as H3K9me2 deposition enzymes G9A and GLP (Maksakova et al., 2013). Hence, we examined the enrichment of H3K9me1/2/3 on HDAC4/5 binding regions. Notably, H3K9me1 was strongly enriched on HDAC4 and HDAC5 binding peaks (Figures S6E and S6F). The H3K9me2/3 were also enriched on HDAC4 and HDAC5 binding peaks (Figures S6E and S6F). In agreement with the enrichment, we observed declining H3K9me1/2 levels, but not that of H3K9me3, after the simultaneous depletion of *Hdac4/5* (Figure 6F). Consistently, total H3K9ac level increased in the absence of HDAC4/5 (Figure 6F). The reduction of H3K9me1/2 was accompanied by the decreased binding of H3K9 methyltransferase G9A to MuERVL (Figure 6G), suggesting that HDAC4/5 affect G9A recruitment. We subsequently found that the levels of H3K9me1/2 declined in *Pim3*^−/−^ ESCs, but H3K9me3 level remained unaltered (Figure 6H). H3K9ac level was also elevated in *Pim3*^−/−^ ESCs (Figure 6H), which was consistent with the exportation of histone deacetylase HDAC4/5 from nucleus. Our ChIP-qPCR results further validated the increment of H3K9ac and the reduction of H3K9me1/2 on MuERVL in *Pim3*^−/−^ ESCs (Figures 6I–6K). Therefore, we conclude that the increment of H3K9ac and decrement of H3K9me1/2 on MuERVL following HDAC4/5 nuclear export may allow the activation of MuERVL in *Pim3*^−/−^ ESCs.

## Discussion

In summary, we propose the following model that in the presence of PIM3, hypo-phosphorylated HDAC4/5 repress MuERVL. In the absence of PIM3, activated AMPK phosphorylates HDAC4/5 and mediates their cytoplasmic localization, thereby causing H3K9ac enrichment and depletion of H3K9me1/2 on MuERVL, and subsequently releasing MuERVL from transcriptional silencing (Figure 6L). It is noteworthy that the retinoic acid signaling pathway and the P53 pathway were recently found to activate the totipotent 2C-like state and MuERVL (Iturbide et al., 2021; Wang et al., 2021). Our study alternatively proves the PIM3 pathway as a suppressive signaling pathway of the 2C-like state in ESCs.

PIM kinases and their impacts on AMPK pathway have been previously reported in cancer cells and somatic cells (Beharry et al., 2011; Han et al., 2019). Consistently, we found that PIM3 was highly expressed among PIM kinases in ESCs (Figure S1A), and its loss triggered AMPK phosphorylation in ESCs (Figure 4H). In mouse embryonic fibroblasts with all of *Pim1/2/3* deleted, overexpression of *Pim3* alone can decrease AMPK activation (Beharry et al., 2011), implicating an important role of PIM3 in regulating AMPK activity. PIM may mediate AMPK activation through LKB1, as phosphorylation of AMPK (Thr172) was insensitive to PIM inhibitors in LKB1-deficient cell lines (Beharry et al., 2011; Hahn-Windgassen et al., 2005). Recently, it was found that PIM mediated LKB1 phosphorylation at Ser334, while LKB1 overexpression was able to rescue PIM inhibitor-caused enhanced AMPK phosphorylation in LKB1-deficient cells (Mung et al., 2021), confirming the role of LKB1 downstream of PIM to activate AMPK. LKB1-AMPK has recently been found to activate an embryonic diapause-like state by repressing mTOR (Hussein et al., 2020). In contrast, inhibition of AMPK hinders the formation of 4-cell embryos and blastocysts (He et al., 2020). The AMPK pathway is also linked to promoting *Nanog* expression during primed to naive state transition (Liu et al., 2021). These findings imply a potential role of AMPK in development and cell potency regulation. It was noticed that *Pim3* loss activated *Nanog*, which was similarly activated in ground state ESCs and expanded potential stem cells (Miyanari and Torres-Padilla, 2012; Silva et al., 2009; Yang et al., 2017). Thus, it is possible that PIM3-downstream AMPK is involved in the potency regulation of other alternative ESC states.

Histone deacetylases HDAC1/2, which are ubiquitously expressed nucleus-localized class I HDACs (Dokmanovic et al., 2007), cooperate with other epigenetic repressors to silence MuERVL and 2C-like state (Guallar et al., 2018; Macfarlan et al., 2011; Yang et al., 2020). However, the deletion of *Hdac1* alone does not induce activation of MuERVL but activates the expression of RLTR45 (Reichmann et al., 2012), implying that HDAC1 may not be the only repressor of MuERVL, and other HDACs may participate in the repression of MuERVL. In complement to previous findings, we identified class IIa HDACs HDAC4/5 as repressors of MuERVL. Different from class I HDACs, class IIa HDACs are known for their ability to shuttle between cytoplasm and nucleus (Dokmanovic et al., 2007), thereby regulating tissue-specific gene expression. Since MuERVL is considered to be a specific 2C marker, its regulation by class IIa HDACs may represent an ESC-specific regulation of 2C-like state. Rather than acting as constitutive repressors, class IIa HDACs HDAC4/5 were exploited by PIM3-AMPK signaling to allow ESCs to enter and exit the 2C-like state dynamically.

Histone deacetylases facilitate the formation of heterochromatin by catalyzing the removal of histone acetylation and allowing the spread of silencing heterochromatin marks such as H3K9 methylation (Allshire and Madhani, 2018). As members of histone deacetylases, HDAC4/5 are able to remove histone actylation of H3K9 and H3K27 (Di Giorgio et al., 2020, 2021; Hou et al., 2020). Consistently, we observed a global increment of H3K9ac after the depletion of both *Hdac4* and *Hdac5* (Figure 6F). This was accompanied by the reduced association of G9A on MuERVL (Figure 6G). Consistently, *Pim3* loss and its associated HDAC4/5 nuclear exportation caused a similar phenotype that the enrichment of H3K9ac increased and H3K9me1/2 declined on MuERVL (Figures 6I–6K). Given that H3K9ac and H3K9me1/2 cannot exist at the same time, it is possible that HDAC4/5-mediated removal of H3K9ac on MuERVL facilitates the deposition of H3K9me1/2 by G9A. In addition, HDAC4 was reported to interact with another histone methyltransferase SUV39H1 (Hohl et al., 2013). The observation that the HDAC complexes contain H3K9 methyltransferases further substantiates this hypothesis (Nakayama et al., 2001).

In conclusion, our study demonstrates that PIM3 represses MuERVL and 2C-like state via AMPK by impeding the phosphorylation and nuclear export of HDAC4/5. Our insights into the role of the PIM3-AMPK-HDAC4/5 axis provide a way to control the 2C-like totipotent state through altering ESC external signaling pathways.

## Experimental procedures

### Cell culture and treatment of small molecules

Mouse E14 and J1 ESCs were cultured on a 12-well tissue culture plate (353043, Falcon) coated with 0.2% gelatin (G1890, Sigma) in standard serum culture conditions supplemented with 1,000 U/mL LIF (Z03077, GenScript). Small molecules were used to treat ESCs for 48 h with the concentration indicated in Table S1.

### Gene knockdown and gene overexpression

For gene knockdown, shRNAs were cloned into the pSuper-puro plasmid, respectively. For the knockdown of *Hdac4* and *Hdac5* at the same time, both *Hdac4* shRNA and *Hdac5* shRNA were cloned into the same pSuper-puro plasmid. ESCs were selected for 72 h with 1 μg/mL puromycin. The transfections were performed using Polyjet transfection reagent (SL100688, SignaGene) according to the manufacturer’s protocol. For gene overexpression, pCAG-3HA-*Hdac4* or *Hdac5* was transfected into WT ESCs or *Pim3*^−/−^ ESCs, which were selected with 800 μg/mL hygromycin B for 2 weeks. The shRNA sequences of the target genes in this study are shown in Table S2.

### Generation of *Pim3*-deficient ESC lines

*Pim3* was knocked out by CRISPR-CAS9 in ESCs. In brief, single guide RNAs (sgRNAs) targeting two distinct regions of the *Pim3* exons were cloned into the PX458 plasmid (Addgene #48138). ESCs were transfected with *Pim3* knockout plasmid by Polyjet for 72 h. Genomic DNA was extracted, and PCR was performed to detect mutation sites using primers flanking sgRNA target regions. Both western blot and DNA sequencing were used to confirm gene knockout. The sequences of sgRNAs and validation primers are shown in Table S2.

### RNA purification and qPCR

The total RNA was isolated from ESCs using RNAiso Plus (9109, TaKaRa). DNase I was used to remove DNA contamination. cDNA was synthesized using 1 μg total RNA with HifairIII First Strand cDNA Synthesis Kit (11139ES60, Yeasen) in RNase-free tube (404001, NEST Biotechnology). Hieff qPCR SYBR Green Master Mix (11202ES08, Yeasen) was used for qPCR on CFX384 Real-Time System (Bio-Rad). The sequences of the primers are shown in Table S2.

### Western blot analysis

ESCs were trypsinized, washed with PBS, and lysed for 30 min on ice in RIPA buffer supplemented with PMSF. Proteins were loaded onto SDS-PAGEs and were transferred to 0.45 μm PVDF membranes, which were blotted with primary antibodies overnight at 4°C (Table S3), followed by incubation with horseradish peroxidase (HRP)-labeled anti-rabbit IgG (sc-2004, Santa Cruz) or anti-mouse IgG (sc-516102, Santa Cruz) for imaging. For phosphorylated protein samples, phosphatase inhibitors (P1081, Beyotime) and protease inhibitors (B14001, Bimake) were added during SDS-PAGE analysis. All western blot results were done at least twice.

### RNA-seq and data analysis

Total RNA was extracted from two biological samples of WT ESCs and two clones of *Pim*3^−/−^ ESCs (clone 1 and clone 2) in RNAiso Plus. Total 4 μg RNA was used for RNA-seq by GENEWIZ. Cutadapt was used to remove adapter sequences and low-quality reads. Hisat2 was used to map RNA-seq data to mm10 genome assembly. Genes were annotated according to the Ensembl database. TEs were annotated according to UCSC Genome Browser (RepeatMasker). The KEGG (Kyoto Encyclopedia of Genes and Genomes) analysis was performed using the RDAVID Web Service. The gene set enrichment analysis (GSEA) of 2C genes and TSC genes was done with gseapy. The Remove Batch Effect function in LIMMA package was used to normalize expression matrix of different experimental groups for PCA clustering analysis.

### Chromatin immunoprecipitation

Chromatin immunoprecipitation assays were performed as described. ESCs cross-linked with 1% formaldehyde were lysed and sonicated. After sonication, the DNA fragments were incubated overnight with the appropriate amount of antibody-loaded (Table S3) protein G MagBeads (L00274, GenScript). The chromatin samples were then eluted and decrosslinked. Immunoprecipitated DNA and input DNA were subjected to qPCR analysis. The gene-specific primers are listed in Table S2.

### ChIP-seq and data analysis

At least 2 ng of ChIP DNA was used for ChIP-seq library preparation. The ChIP-seq libraries were constructed with ATseq kit according to the manufacturer’s protocols and were sequenced by Novogene Corporation. For ChIP-seq data analysis, the adapter sequences and reads with Phred score < 5 were removed with Cutadapt, and the data were mapped to the mouse mm10 genome assembly using Bowtie2. ChIP-seq signal enrichment, signal heatmaps, and line plots were generated by Deeptools. HDAC4 and HDAC5 binding peaks were predicted by Macs2. The centers of MuERVL were inferred from RepeatMasker.

### Extraction of cytoplasmic and nuclear proteins

Cytoplasmic and nuclear extracts were performed as previously described. In brief, ESCs (2 × 10^6^ cells) were harvested and lysed in 200 μL lysis buffer containing protease inhibitors for 5 min on ice. The supernatant after centrifugation was collected as the cytoplasmic extract, and the precipitate was used as the nuclear extract. The nuclear proteins were extracted with RIPA buffer supplemented with PMSF. The cytoplasmic and nuclear extracts were loaded onto SDS-PAGE and analyzed by western blot.

### Statistics

qPCR results and summary of flow cytometry results were analyzed by the two-sided Student’s t test in Microsoft Excel. Significant differences were defined as ^∗^p < 0.05 or ^∗∗^p < 0.01. The Wald test was used to determine differentially expressed genes and TEs. The Kolmogorov-Smirnov statistic was used in GSEA analysis to determine false discovery rate and p value.

## Author contributions

Conceptualization: X.L.; methodology: X.L., Xin Zhao, and J.S.; investigation: Xin Zhao, J.S., M.Z., and W.Z.; visualization: Xin Zhao, J.S., Xuan Zhao, and X.F.; supervision: X.L.; writing: X.L., Xin Zhao, J.S., and Xuan Zhao.

## Data Availability

The sequencing datasets produced in this study are available in the Gene Expression Omnibus (GEO) database: RNA-seq data after *Pim3* deletion, GEO: GSE178129, and ChIP-seq data for HDAC4 and HDAC5, GEO: GSE178130. Published data analyzed in this study are available in GEO under accession numbers GSE121451 (RNA-seq after *Dux* induction) (Fu et al., 2019), GSE133234 (RNA-seq after *Dux* withdrawal) (Fu et al., 2020), GSE54412 (H3K9me1/2 ChIP-seq) (Liu et al., 2015), and GSE77440 (H3K9me3 ChIP-seq) (Riso et al., 2016).

## Supplemental information

Document S1. Figures S1–S6 and Tables S1–S3

Data S1 and S2. Differentially expressed genes after Pim3 knockout

Document S2. Article plus supplemental information
