# Supplementary material for: PIM3-AMPK-HDAC4/5 axis restricts MuERVL-marked 2-cell-like state in embryonic stem cells
Source: Stem Cell Reports. 2022 Sep 22;17(10):2256–71. doi: 10.1016/j.stemcr.2022.08.009 (PMC9561635; doi:10.1016/j.stemcr.2022.08.009)
Supplement: Document S1. Figures S1–S6 and Tables S1–S3 [file mmc1.pdf]

**Stem Cell Reports, Volume *17***

## Supplemental Information

# PIM3-AMPK-HDAC4/5 axis restricts MuERVL-marked 2-cell-like state in embryonic stem cells

**Xin Zhao, Jian Shen, Xuan Zhao, Miao Zhang, Xiao Feng, Weiyu Zhang, and Xinyi Lu**

## Supplemental information

# PIM3-AMPK-HDAC4/5 axis restricts MuERVL-marked 2-cell-like state in embryonic stem cells

Xin Zhao, Jian Shen, Xuan Zhao, Miao Zhang, Xiao Feng, Weiyu Zhang, Xinyi Lu

## SUPPLEMENTAL FIGURES

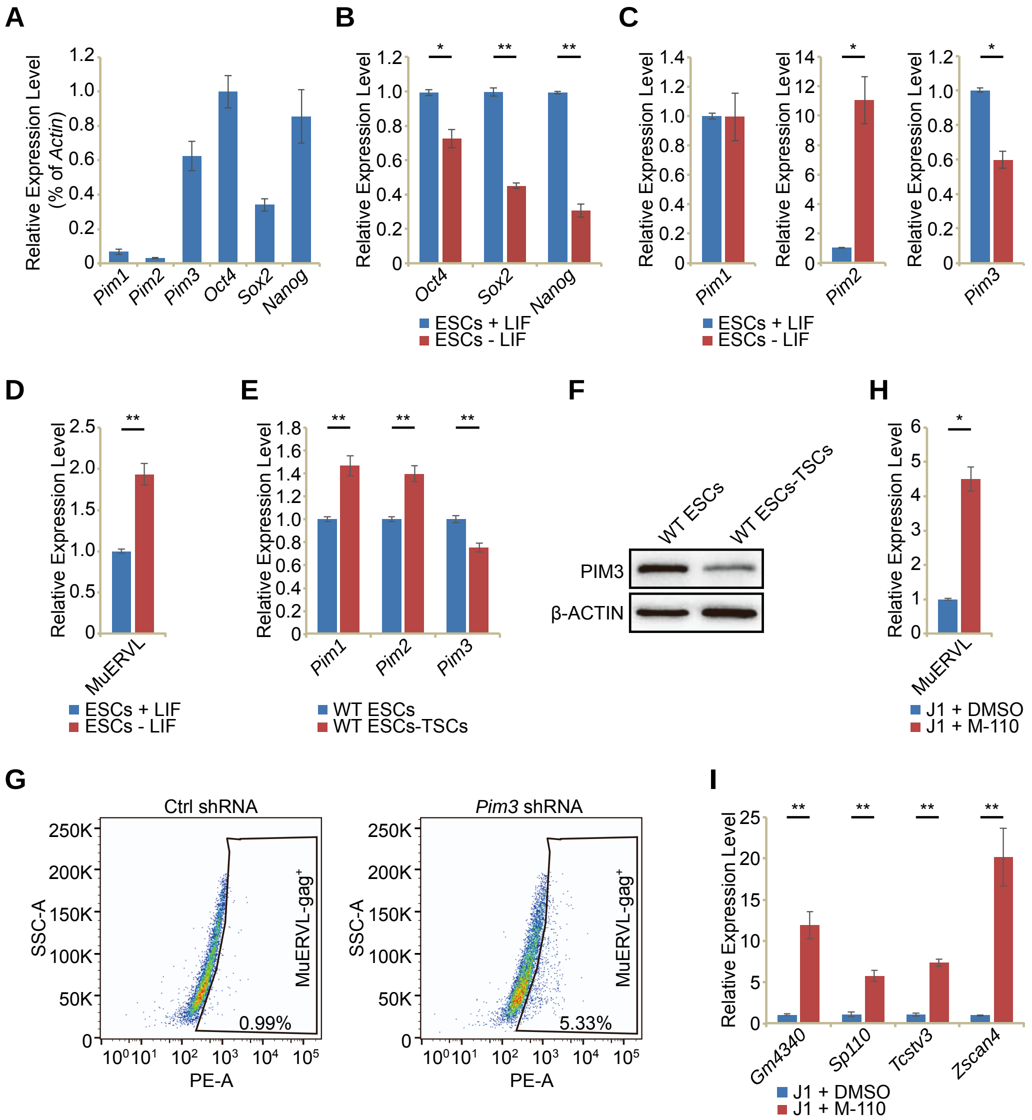


**Figure S1. *Pim3* expression and validation of *Pim3* function, related to Figure 1.**

**(A)** qPCR analysis of PIM kinases and pluripotency genes expression in ESCs. Data are shown as mean ± standard error of the mean (s.e.m.) of three independent experiments (n = 3).

**(B)** qPCR analysis of pluripotency genes (*Oct4*, *Sox2* and *Nanog*) expression in ESCs differentiation induced by LIF withdrawal. ESCs cultured in the presence of LIF (ESCs + LIF) were used as a control. The data are presented as mean ± s.e.m. (n = 3 independent experiments).

**(C)** The expression levels of PIM kinases in ESCs before and after LIF-withdrawal induced differentiation, as measured by RT-qPCR. Data are shown as mean ± s.e.m. (n = 3 independent experiments).

**(D)** The expression level of MuERVL in ESCs before and after LIF-withdrawal induced differentiation, as measured by RT-qPCR. Data are shown as mean ± s.e.m. (n = 3 independent experiments).

**(E)** qPCR analysis of the expression of PIM kinases in WT ESCs differentiated into TSCs. Data are shown as mean ± s.e.m. (n = 3 independent experiments).

**(F)** Western blot analysis of PIM3 protein after directed differentiation of WT ESCs towards TSCs. β-

ACTIN was used as a loading control.

**(G)** Flow cytometry scatter diagram analysis of the MuERVL-gag$^+$ population in ESCs treated with control (Ctrl) shRNA or *Pim3* shRNA.

**(H)** qPCR analysis of the expression of MuERVL in J1 ESCs treated with M-110 or DMSO. DMSO treated samples were included as controls. Data are presented as mean ± s.e.m. (n = 3 independent experiments).

**(I)** qPCR analysis of the expression of 2-cell embryo genes in J1 ESCs treated with M-110 or DMSO. Data are presented as mean ± s.e.m. (n = 3 independent experiments). *$p < 0.05$, **$p < 0.01$ in Student's *t*-test.

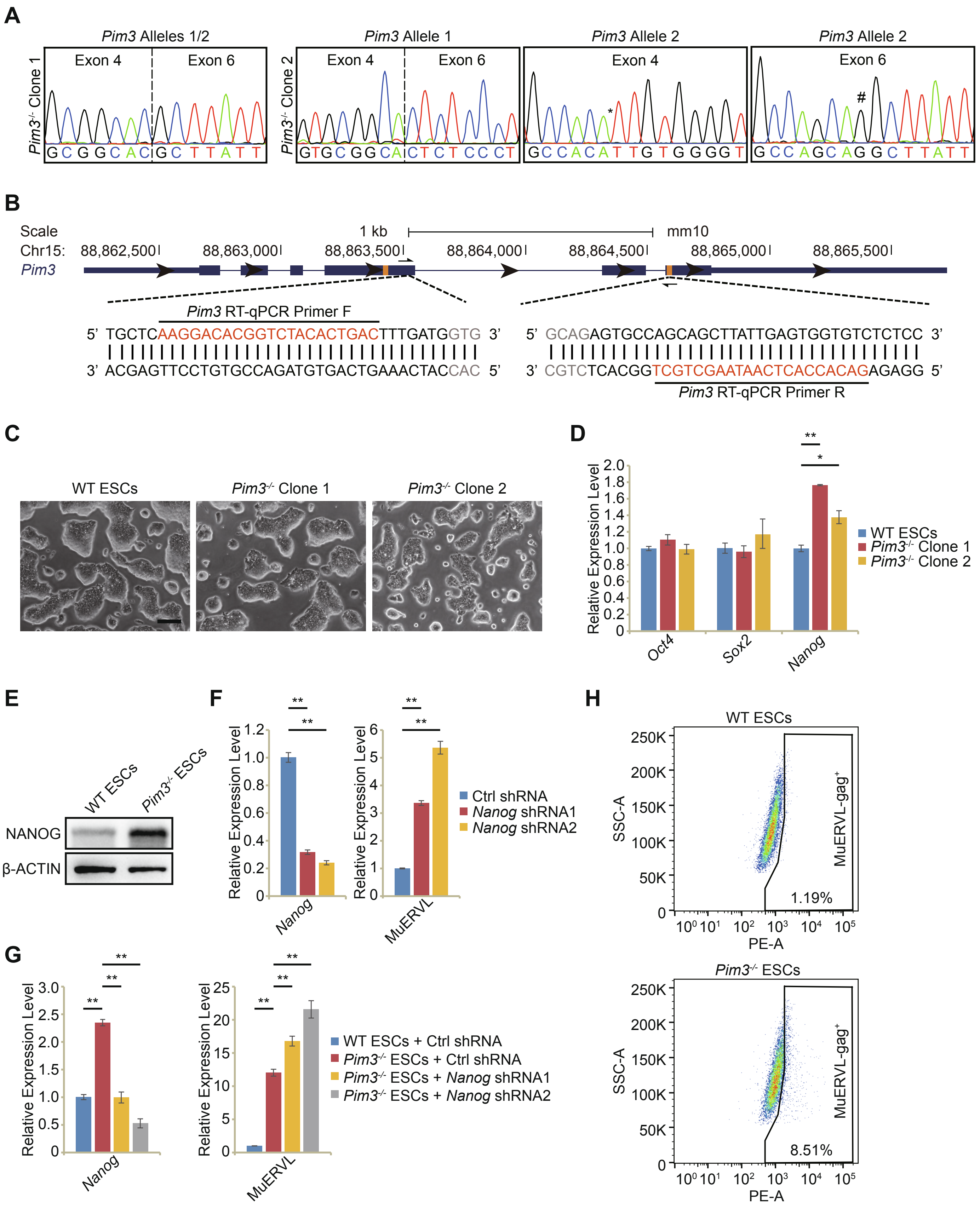
A
Pim3 Alleles 1/2
Pim3 Allele 1
Pim3 Allele 2
Pim3 Allele 2
Exon 4
Exon 6
Pim3-/- Clone 1
Pim3-/- Clone 2
B
Scale
1 kb
mm10
Chr15: 88,862,500 88,863,000 88,863,500 88,864,000 88,864,500 88,865,000 88,865,500
Pim3
Pim3 RT-qPCR Primer F
5' TGCTCAAGGACACGGTCTACACTGACTTTGATGGTG 3'
3' ACGAGTTCCTGTGCCAGATGTGACTGAAACTACCAC 5'
5' GCAGAGTGCCAGCAGCTTATTGAGTGGTGTCTCTCC 3'
3' CGTCTCACGGTCGTCGAATAACTCACCACAGAGAGG 5'
Pim3 RT-qPCR Primer R
C
WT ESCs
Pim3-/- Clone 1
Pim3-/- Clone 2
D
Relative Expression Level
Oct4
Sox2
Nanog
WT ESCs
Pim3-/- Clone 1
Pim3-/- Clone 2
E
WT ESCs
Pim3-/- ESCs
NANOG
β-ACTIN
F
Relative Expression Level
Nanog
MuERVL
Ctrl shRNA
Nanog shRNA1
Nanog shRNA2
G
Relative Expression Level
Nanog
MuERVL
WT ESCs + Ctrl shRNA
Pim3-/- ESCs + Ctrl shRNA
Pim3-/- ESCs + Nanog shRNA1
Pim3-/- ESCs + Nanog shRNA2
H
WT ESCs
Pim3-/- ESCs
SSC-A
PE-A
MuERVL-gag+
1.19%
8.51%

**Figure S2. Confirmation of *Pim3* knockout in ESCs, related to Figure 2.**

**(A)** DNA sequencing results of mutation sites in two *Pim3*$^{-/-}$ ESC clones. The asterisk indicates the deletion of a base at this location. The hash indicates the insertion of a base at this location.

**(B)** Schematic of the location of *Pim3* RT-qPCR primers. The sgRNA target sequences on exon 4 and exon 6 of *Pim3* are highlighted in orange. The grey letters represent intron sequences.

**(C)** Cell morphology of WT ESCs and *Pim3*$^{-/-}$ ESCs. Scale bar, 100 μm.

**(D)** qPCR analysis of the expression of pluripotency markers in WT ESCs and *Pim3*$^{-/-}$ ESCs. Biological triplicate data (n = 3 independent experiments) are presented as mean ± s.e.m.

**(E)** Western blot analysis of NANOG protein in WT ESCs and *Pim3*$^{-/-}$ ESCs. β-ACTIN was used as a loading control.

**(F)** qPCR analysis of the expression of *Nanog* and MuERVL after the depletion of *Nanog* in WT ESCs. Data are presented as mean ± s.e.m. (n = 3 independent experiments).

**(G)** qPCR analysis of the expression of *Nanog* and MuERVL after the depletion of *Nanog* in *Pim3*$^{-/-}$ ESCs. Data are presented as mean ± s.e.m. (n = 3 independent experiments). *$p < 0.05$, **$p < 0.01$ in Student's *t*-test.

**(H)** Flow cytometry analysis of the MuERVL-gag$^{+}$ population within WT ESCs or *Pim*3$^{-/-}$ ESCs.

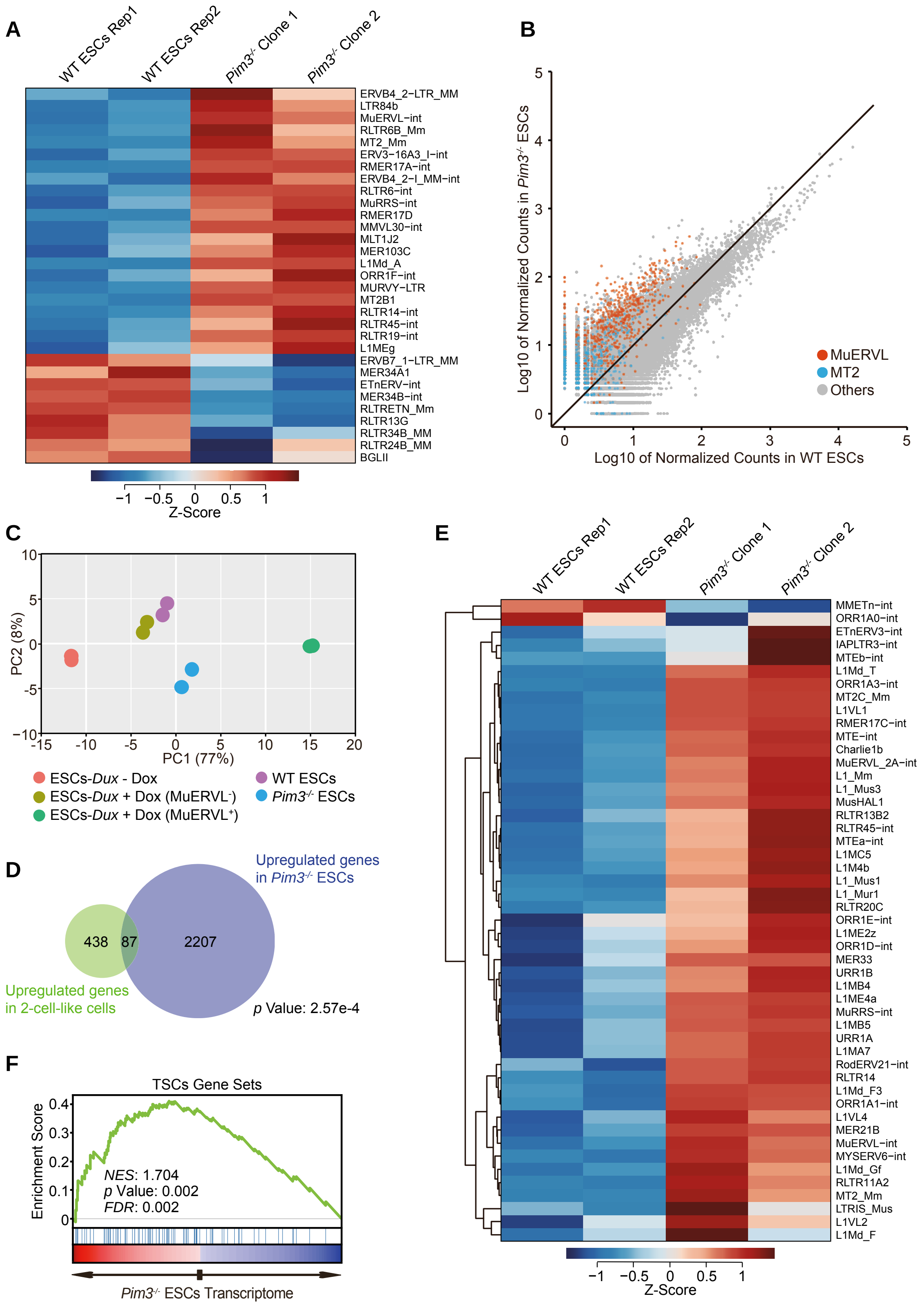
A
WT ESCs Rep1
WT ESCs Rep2
Pim3-/- Clone 1
Pim3-/- Clone 2
ERVB4_2-LTR_MM
LTR84b
MuERVL-int
RLTR6B_Mm
MT2_Mm
ERV3-16A3_I-int
RMER17A-int
ERVB4_2-I_MM-int
RLTR6-int
MuRRS-int
RMER17D
MMVL30-int
MLT1J2
MER103C
L1Md_A
ORR1F-int
MURVY-LTR
MT2B1
RLTR14-int
RLTR45-int
RLTR19-int
L1MEg
ERVB7_1-LTR_MM
MER34A1
ETnERV-int
MER34B-int
RLTRETN_Mm
RLTR13G
RLTR34B_MM
RLTR24B_MM
BGLII
Z-Score
B
Log10 of Normalized Counts in Pim3-/- ESCs
Log10 of Normalized Counts in WT ESCs
MuERVL
MT2
Others
C
PC2 (8%)
PC1 (77%)
ESCs-Dux - Dox
ESCs-Dux + Dox (MuERVL-)
ESCs-Dux + Dox (MuERVL+)
WT ESCs
Pim3-/- ESCs
D
Upregulated genes in Pim3-/- ESCs
438
87
2207
Upregulated genes in 2-cell-like cells
p Value: 2.57e-4
E
WT ESCs Rep1
WT ESCs Rep2
Pim3-/- Clone 1
Pim3-/- Clone 2
MMETn-int
ORR1A0-int
ETnERV3-int
IAPLTR3-int
MTEb-int
L1Md_T
ORR1A3-int
MT2C_Mm
L1VL1
RMER17C-int
MTE-int
Charlie1b
MuERVL_2A-int
L1_Mm
L1_Mus3
MusHAL1
RLTR13B2
RLTR45-int
MTEa-int
L1MC5
L1M4b
L1_Mus1
L1_Mur1
RLTR20C
ORR1E-int
L1ME2z
ORR1D-int
MER33
URR1B
L1MB4
L1ME4a
MuRRS-int
L1MB5
URR1A
L1MA7
RodERV21-int
RLTR14
L1Md_F3
ORR1A1-int
L1VL4
MER21B
MuERVL-int
MYSERV6-int
L1Md_Gf
RLTR11A2
MT2_Mm
LTRIS_Mus
L1VL2
L1Md_F
Z-Score
F
TSCs Gene Sets
Enrichment Score
NES: 1.704
p Value: 0.002
FDR: 0.002
Pim3-/- ESCs Transcriptome

**Figure S3. Transition of *Pim3*$^{-/-}$ cells towards 2-cell-like cells, related to Figure 3.**

**(A)** Expression heatmap of selected retrotransposons after *Pim3* knockout (fold change ≥ 2; Wald test, adjusted $p < 0.05$).

**(B)** Dot plot of all TEs in WT ESCs and *Pim*3$^{-/-}$ ESCs. Red dots represent MuERVL; blue dots represent MT2.

**(C)** Principal component analysis (PCA) based on gene expression in WT ESCs, *Pim*3$^{-/-}$ ESCs and Dox-induced *Dux*-driving the entry of ESCs to 2C-like cells (MuERVL$^{+}$). WT, wild type. -, negative, +, positive.

**(D)** Venn diagram showing overlapped number between upregulated genes in *Pim*3$^{-/-}$ ESCs and genes enriched in 2-cell-like cells (fold change ≥ 1.5; Wald test, adjusted $p < 0.05$).

**(E)** Expression heatmap of retrotransposons that up-regulated in 2-cell embryos after *Pim3* knockout. List of retrotranposons that were activated in 2-cell embryos was referenced from Macfarlan et al., 2012.

**(F)** GSEA of TSC genes in the transcriptome of *Pim*3$^{-/-}$ ESCs. Red, up-regulated genes; blue, down-regulated genes; *NES*, normalized enrichment scores; *FDR*, false discovery rate.

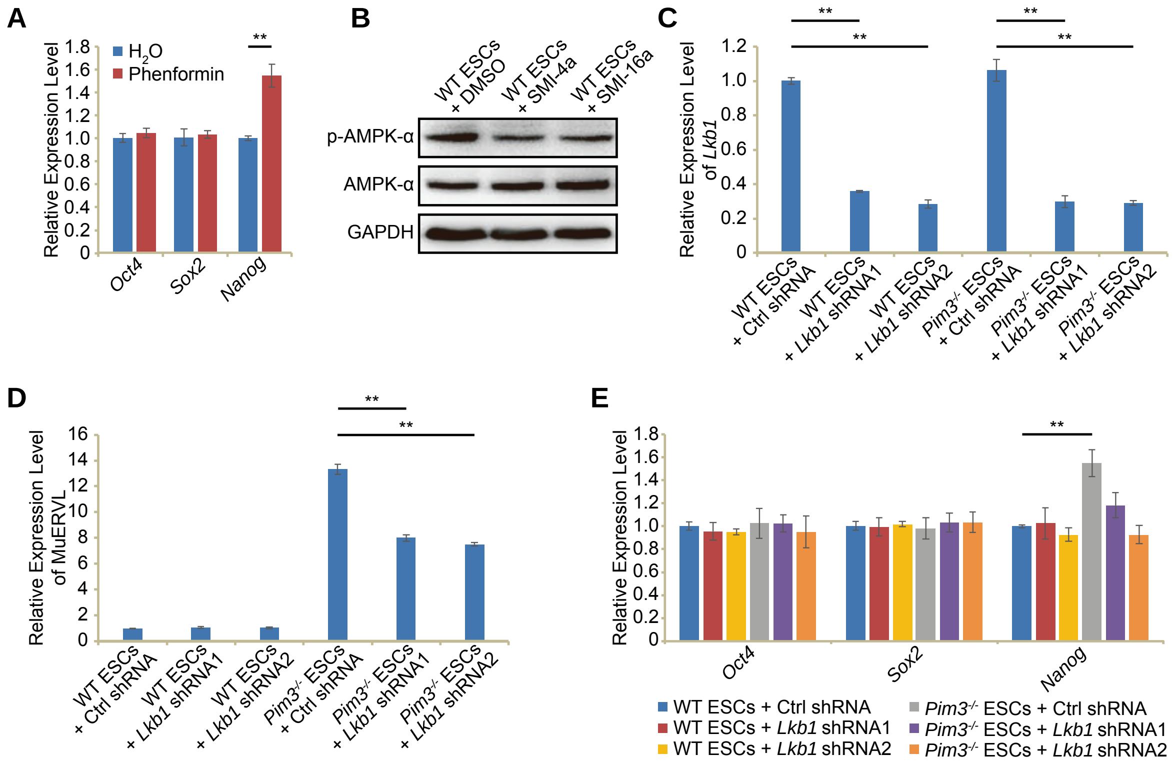


**Figure S4. Rescue phenotype of *Pim*3$^{-/-}$ ESCs by *Lkb1* depletion, related to Figure 4.**

**(A)** qPCR analysis of the expression of pluripotency markers in control ESCs and ESCs treated with 1.5 mM phenformin. Data are presented as mean ± s.e.m. (n = 3 independent experiments).

**(B)** Western blot analysis of p-AMPK-α and AMPK-α in WT ESCs treated with SMI-4a or SMI-16a. DMSO-treated sample was included as a control. GAPDH was used as a loading control.

**(C)** qPCR analysis of the expression of *Lkb1* after *Lkb1* depletion in WT ESCs and *Pim*3$^{-/-}$ ESCs. Data are presented as mean ± s.e.m. (n = 3 independent experiments).

**(D)** qPCR analysis of the expression of MuERVL after *Lkb1* depletion in WT ESCs and *Pim*3$^{-/-}$ ESCs. Data (n = 3 independent experiments) are presented as mean ± s.e.m.

**(E)** qPCR analysis of the expression of pluripotency markers after *Lkb1* depletion in WT ESCs and *Pim*3$^{-/-}$ ESCs. Data (n = 3 independent experiments) are presented as mean ± s.e.m. **$p < 0.01$ in Student's *t*-test.

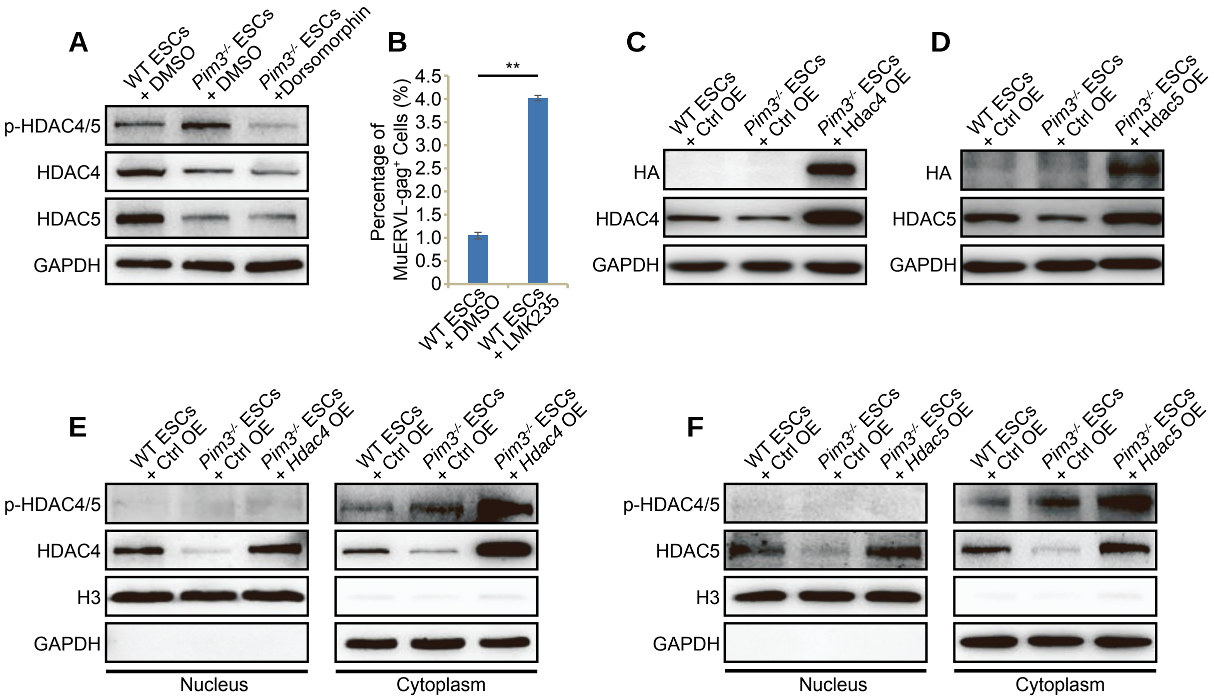


**Figure S5. Overexpression of *Hdac4/5* in *Pim*3$^{-/-}$ ESCs, related to Figure 5.**

**(A)** Western blot analysis of HDAC4/5 and p-HDAC4/5 levels in *Pim*3$^{-/-}$ ESCs treated with dorsomorphin. DMSO treated samples were included as a control. GAPDH was used as a loading control.

**(B)** Flow cytometry analysis of the MuERVL-gag$^{+}$ population in WT ESCs treated with DMSO and LMK-235 respectively. DMSO-treated sample was included as control. Data are presented as mean ± s.e.m. (n = 3 independent experiments). $^{**}p < 0.01$ in Student's *t*-test.

**(C)** Immunoblot analysis of the expression of HDAC4 after overexpression of *Hdac4* in *Pim*3$^{-/-}$ ESCs. GAPDH was included as a loading control.

**(D)** Immunoblot analysis of the expression of HDAC5 after overexpression of *Hdac5* in *Pim*3$^{-/-}$ ESCs. GAPDH was included as a loading control.

**(E)** Western blot analysis of HDAC4 and p-HDAC4/5 expression after *Hdac4* overexpression (OE) in the nucleus and cytoplasm of *Pim*3$^{-/-}$ ESCs. GAPDH was used as a loading control to the cytoplasm. H3 was used as a loading control to the nucleus.

**(F)** Western blot analysis of HDAC5 and p-HDAC4/5 expression after *Hdac5* overexpression (OE) in the nucleus and cytoplasm of *Pim*3$^{-/-}$ ESCs. GAPDH was used as a loading control to the cytoplasm. H3 was used as a loading control to the nucleus.

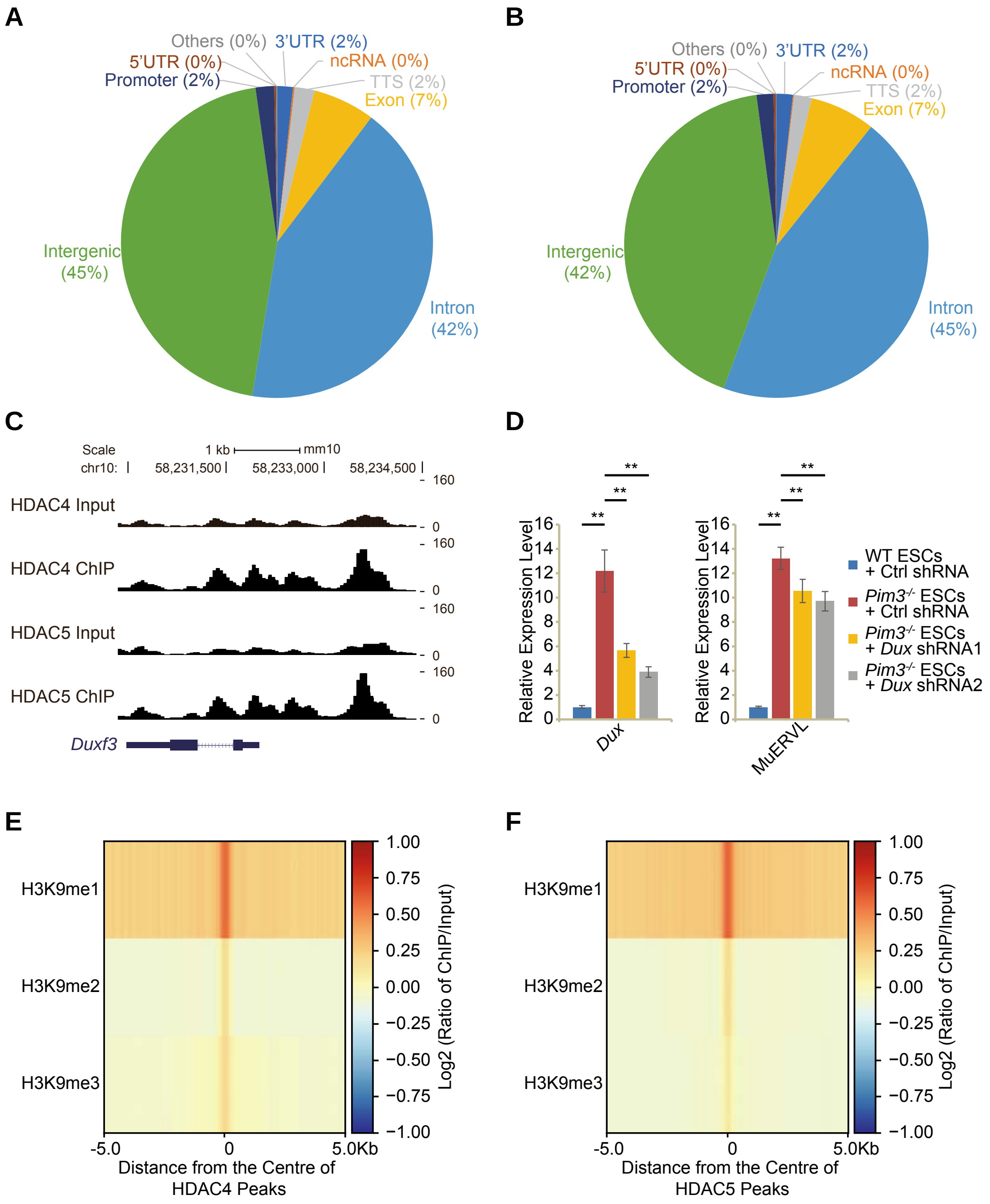


**Figure S6. Enrichment of H3K9 methylation on HDAC4/5 binding regions, related to Figure 6.**

**(A-B)** Locations of HDAC4 (A) and HDAC5 (B) peaks relative to the nearest transcription units (promoter, -1 kb to +100 bp around transcriptional start sites). Homer was used to annotate the location of peaks in terms of important genomic features.

**(C)** ChIP-seq tracks for the enrichment of HDAC4 and HDAC5 on *Dux* promoter region in WT ESCs.

**(D)** qPCR analysis of the expression of *Dux* and MuERVL after the depletion of *Dux* in *Pim*3$^{-/-}$ ESCs. Data are presented as mean ± s.e.m. (n = 3 independent experiments). WT, wild type; ***p* < 0.01 in Student's *t*-test.

**(E-F)** The heatmaps of published H3K9me1/2/3 enrichment on HDAC4 (E) and HDAC5 (F) binding peaks in wild type ESCs after normalization of ChIP-seq signal against input. H3K9me1/2 ChIP-seq data in WT ESCs were obtained from GSE54412; H3K9me3 ChIP-seq data in WT ESCs were obtained from GSE77440.

## SUPPLEMETNAL TABLES

**Table S1. Small molecule inhibitors/activators used in the manuscript.**

| Name | Function | Catalog Number | Final concentration |
|---|---|---|---|
| SMI-4a | PIM1 inhibitor | S47174, MedMol | 10 μM |
| SMI-16a | PIM1/2 inhibitor | S6497, Selleck Chemical | 10 μM |
| M-110 | PIM3 inhibitor | HY-12830, MedChemExpress | 2 μM |
| SB203580 | MAPK inhibitor | 20200210, Xiyashiji | 15 μM |
| PD0325901 | ERK inhibitor | T6189, TargetMol | 1 μM |
| Rapamycin | mTORC1 inhibitor | S115842, Aladdin | 100 nM |
| Phenformin | AMPK activator | S2542, Selleck Chemical | 1.5 mM |
| Dorsomorphin | AMPK inhibitor | T1977, TargetMol | 1 μM |
| CHIR-99021 | GSK3β inhibitor | IWN1011, GeneOperation | 3 μM |
| LMK-235 | HDAC4/5 inhibitor | T6061, TargetMol | 3 μM |

**Table S2. Sequences of primers, shRNAs and gRNAs used in the manuscript.**

| **Gene** | **Sequence F** | **Sequence R** |
|---|---|---|
| *Pim1* | CTGGAGTCGCAGTACCAGG | CAGTTCTCCCCAATCGGAAATC |
| *Pim2* | TTCAGCGGGCTCAATATACGC | CCAAGTCGGTATTCGGCCTC |
| *Pim3* | AAGGACACGGTCTACACTGAC | GACACCACTCAATAAGCTGCT |
| *Oct4* | GTGGAAAGCAACTCAGAGG | GGTTCCACCTTCTCCAACT |
| *Sox2* | GCGGAGTGGAAACTTTTGTCC | CGGGAAGCGTGTACTTATCCTT |
| *Nanog* | TTGCTTACAAGGGTCTGCTACT | ACTGGTAGAAGAATCAGGGCT |
| MuERVL | CTCTACCACTTGGACCATATGAC | GAGGCTCCAAACAGCATCTCTA |
| MER89 | AAGCTTTCCCACTCCTCTGC | CCAAATGAGAACAAGCAAAG |
| RLTR1B-int | AAGAATTTTCTTGCTTGCCTTG | TAAAGAGGCTCAGCTGTGTCAG |
| RLTR6 | AGTCCCCAGAAACTCACGTA | CTCGAAACCCTCAGCTTACC |
| RLTR45-int | CAAGGTTTAAATGCACAAGCAA | GATACCACCAAGTTCCTGCTTC |
| IAPEY-LTR | TCGGGGTGTAATAGTGGGGT | CGGCAAGAAAGACGCAACAA |
| ERVB4-2-LTR | ACCTTGGACCTTTCTCAATACAT | GACCTCCTCCTAATAACCAAATG |
| MT2B2 | GTACACACCTTTAATCTGGGCC | CAACTAACCCAACAATGGTCAG |
| LINE1 | GGACCAGAAAAGAAATTCCTCCCG | CTCTTCTGGCTTTCATAGTCTCTGG |
| SINE B1 | GTGGCGCACGCCTTTAATC | GACAGGGTTTCTCTGTGTAG |
| major satellite | GACGACTTGAAAAATGACGAAATC | CATATTCCAGGTCCTTCAGTGTGC |
| MuERVL-int | ACCAGTTGCAGAAACGAGGA | GCCACTACAATCCACCCCTT |
| *Dux* | CACTCGACTCACCTCGCTAC | CCATCCGACCCTTGTGACG |
| *Gm4340* | TTGTTGGGAATTTGGCTGCC | CATGGGTGAAAGCTGGCCTA |
| *Sp110* | ATGAAGGTGAACATCGCCTATG | GGACAGAGGGACCAGATTTTG |
| *Spz1* | CCCTTGCAAACTTTGGTACC | TTGGGGAGCCTTTTGATGAT |
| *Tcstv3* | GATCCTGCATCTTATAGTGCCA | TGACTTTCTCACTTCTGGCG |
| *Zfp352* | AAGTCCCACATCTGAAGAAACAC | GGGTATGAGGATTCACCCACA |
| *Zscan4* | GAGATTCATGGAGAGTCTGACTGATGAGTG | GCTGTTGTTTCAAAAGCTTGATGACTTC |
| *Lkb1* | TTGGGCCTTTTCTCCGAGG | CAGGTCCCCCATCAGGTACT |
| *Hdac4* | CACTGCATTTCCAGCGATCC | AAGACGGGGTGGTTGTAGGA |
| *Hdac5* | AGCACCGAGGTAAAGCTGAG | GCTGTGGGAGGGAATGGTT |
| *Gapdh* | AGAAACCTGCCAAGTATGATGAC | GTCATTGAGAGCAATGCCAG |
| *Actb* | GGCTGTATTCCCCTCCATCG | CCAGTTGGTAACAATGCCATGT |
| **Gene** | **shRNA Sequence** | **guide RNA (gRNA) Sequence** |
| *Pim1* | GCAAGACCTCTTCGACTTT | *Pim3* sgRNA 1 |
| *Pim2* | AGGACCAGCATGAAGCCTT | CAATTGTGGGGTCGTGCACC |
| *Pim3* | CAGGACCTCTTCGACTTCAT | *Pim3* sgRNA 2 |
| *Nanog* | shRNA1: GTTAAGACCTGGTTTCAAA | GCAGCTTATTGAGTGGTGTC |
| *Nanog* | shRNA2: GGGAAAGCCATGCGCATTT | |
| *Lkb1* | shRNA1: GGGTCACACTTTACAACAT | |
| *Lkb1* | shRNA2: GAGGACGGCATTATCTACA | |
| *Hdac4* | shRNA1: CATGGGTTTCTGCTACTTTAA | |
| *Hdac4* | shRNA2: GGTTATGCCTATCGCAAAT | |
| *Hdac5* | shRNA1: CCGTAGCCATCACAGCTAAAC | |
| *Hdac5* | shRNA2: CCAAACCAGTTCAGCCTCTAT | |
| *Dux* | shRNA1: CTGGATGAAGTCCAAGTAGAA | |
| *Dux* | shRNA2: GGTTCCCAGGACAGCTTACT | |

**Table S3. Primary antibodies used in current study.**

| **Primary antibodies** | **Catalog Number** |
|---|---|
| Anti-PIM3 | D17C9, Cell Signaling Technology |
| Anti-PIM1 | C93F2, Cell Signaling Technology |
| Anti-PIM2 | D1D2, Cell Signaling Technology |
| Anti-NANOG | ab80892, Abcam |
| Anti-MuERVL-gag | A-2801, EpiGentek |
| Anti-AMPK-α | D5A2, Cell Signaling Technology |
| Anti-p-AMPK-α | 40H9, Cell Signaling Technology |
| Anti-HDAC4 | D8T3Q, Cell Signaling Technology |
| Anti-HDAC5 | D1J7V, Cell Signaling Technology |
| Anti-p-HDAC4/5 | D27B5, Cell Signaling Technology |
| Anti-HA | 30701ES60, Yeasen |
| Anti-H3K9me1 | ab176880, Abcam |
| Anti-H3K9me2 | ab32521, Abcam |
| Anti-H3K9me3 | ab8898, Abcam |
| Anti-H3K9ac | ab32129, Abcam |
| Anti-G9A | ab185050, Abcam |
| Anti-GAPDH | KM9002, Sungenebiotech |
| Anti-β-ACTIN | KM9001, Sungenebiotech |
| Anti-H3 | 17168-1-AP, Proteintech |

## SUPLEMENTAL EXPERIMENTAL PROCEDURES

### ESC differentiation

ESCs were differentiated towards trophoblast stem cells (TSCs) as previously reported (Abad et al., 2013). WT ESCs and *Pim3*$^{-/-}$ ESCs were seeded on gelatin-coated 6-well plate in ESC medium, and 24 h post-treatment, medium was changed to TSC differentiation medium, which contains: Roswell Park Memorial Institute (RPMI) 1640 medium (01-100-1ACS, Biological Industries) supplemented with 20% FBS (SH30070.03, Hyclone), 1 mM pyruvate (SP0100, Solarbio), 2 mM L-glutamine (G0200, Solarbio), 1% Penicillin/Streptomycin (P1400, Solarbio), 0.1 mM β-mercaptoethanol (M3148-250, Sigma), 25 ng/mL FGF4 (Z02984, GenScript) and 1 μg/mL heparin (S12004, Yuanye Bio-Technology). The medium was refreshed daily to maintain TSCs for 3 days. For differentiation of ESCs by LIF withdrawal, $1.0 \times 10^5$ ESCs were cultured in standard serum culture conditions on a well of the 12-well tissue culture plate without LIF for 4 days. The other components of the medium were the same as previously described.

### Immunostaining and flow cytometry analysis

For flow cytometry analysis after immunostaining, ESCs were harvested and fixed in cold 80% ethanol at 4 °C. The cells were permeated by 0.5% Triton X-100 for 30 min and blocked with 1% bovine serum albumin (BSA) for 30 min. Next, cells were stained with the MuERVL-gag primary antibody (A-2801, EpiGentek) at 4 °C for 2 h, and subsequently stained for 1-1.5 h at 4 °C with the secondary antibody Alexa Fluor 594-conjugated goat anti-rabbit IgG (ZF-0516, ZSGB-BIO). The percentage of MuERVL-gag$^+$ cells was detected by flow cytometry (BD LSRFortessa).

### Protein immunoprecipitation

Cells were lysed in lysis buffer (20 mM Tris-HCl pH = 7.5, 150 mM NaCl, 20 mM KCl, 1.5 mM $MgCl_2$, 1% Glycine, and 0.5% TritonX-100) supplemented with protease inhibitors (B14001, Bimake) and phosphatase inhibitors (P1081, Beyotime) on ice for 30 minutes. After centrifugation at 13,000 rpm for 20 minutes, the supernatant was collected and precleared. Lysates were respectively incubated with

anti-HDAC4 (D8T3Q, Cell Signaling Technology) or anti-HDAC5 (D1J7V, Cell Signaling Technology) antibody-loaded protein G MagBeads (L00274, GenScript) at 4°C overnight. The beads were washed three times with lysis buffer, and the bound proteins were released from the beads by boiling in 5 × loading buffer for 5 minutes. The western blot was performed to detect the proteins in the immunoprecipitated samples.

## REFERENCES

Abad, M., Mosteiro, L., Pantoja, C., Canamero, M., Rayon, T., Ors, I., Grana, O., Megias, D., Dominguez, O., Martinez, D., et al. (2013). Reprogramming in vivo produces teratomas and iPS cells with totipotency features. Nature *502*, 340-345. 10.1038/nature12586.

Macfarlan, T.S., Gifford, W.D., Driscoll, S., Lettieri, K., Rowe, H.M., Bonanomi, D., Firth, A., Singer, O., Trono, D., and Pfaff, S.L. (2012). Embryonic stem cell potency fluctuates with endogenous retrovirus activity. Nature 487, 57-63. 10.1038/nature11244.
